# Fingolimod-Associated Macular Edema in the Treatment of Multiple Sclerosis

**DOI:** 10.7759/cureus.41520

**Published:** 2023-07-07

**Authors:** Asma A Khan, Sai Dheeraj Gutlapalli, Mehvish Sohail, Priyansh Patel, Sidharth Midha, Surmai Shukla, Divyanshu Dhamija, Adedamola O Bello, Abeer O Elshaikh

**Affiliations:** 1 Medical School, California Institute of Behavioral Neurosciences & Psychology, Fairfield, USA; 2 Internal Medicine, Richmond University Medical Center Affiliated with Mount Sinai Health System and Icahn School of Medicine, New York, USA; 3 Internal Medicine Clinical Research, California Institute of Behavioral Neurosciences & Psychology, Fairfield, USA; 4 Medicine, California Institute of Behavioral Neurosciences & Psychology, Fairfield, USA; 5 Internal Medicine, California Institute of Behavioral Neurosciences & Psychology, Fairfield, USA; 6 Internal Medicine, Baroda Medical College, Vadodara, IND; 7 Radiology, Bharati Vidyapeeth University, Pune, IND; 8 Radiology, California Institute of Behavioral Neurosciences & Psychology, Fairfield, USA; 9 Medicine and Surgery, Qingdao University College of Medical Science, Qingdao, CHN; 10 Internal Medicine, Government Medical College Amritsar, Amritsar, IND; 11 General Medicine, California Institute of Behavioral Neurosciences & Psychology, Fairfield, USA; 12 Psychiatry, St. Martinus University Faculty of Medicine, Willemstad, USA; 13 Psychiatry, California Institute of Behavioral Neurosciences & Psychology, Fairfield, USA; 14 Internal Medicine/Family Medicine, California Institute of Behavioral Neurosciences & Psychology, Fairfield, USA

**Keywords:** fame, adverse effects, multiple sclerosis, macular edema, fingolimod

## Abstract

Multiple sclerosis is a neurological disorder categorized by inflammatory processes with a high prevalence worldwide. It affects both motor and sensory pathways and is also associated with the visual pathway. Fingolimod is a commonly used drug for relapsing-remitting multiple sclerosis. It is a sphingosine 1-phosphate modulator acting on its receptors for immune cell accumulation, neuronal function, embryological development, vascular permeability, smooth muscle cell function, and endothelial barrier maintenance. This review aims to understand the processes, mechanisms, risks, and management of fingolimod-associated macular edema. Due to the anti-inflammatory properties of fingolimod, it decreases various cytokines, including interleukin (IL)-1B and IL-6, spike wave, and spike amplitude, in electrophysiological activities and decreases insoluble receptors for advanced glycation end product ligand. A daily dosage of 0.5 mg of fingolimod has an increased association with macular edema. The serious adverse events of fingolimod are lymphopenia, cardiovascular events, ocular events, and carcinoma. Fingolimod decreases brain volume and increases vascular permeability, resulting in increased macular volume and damage to the blood-retinal barrier, which causes an increased risk for macular edema. Cystoid macular edema is more common in older individuals suffering from comorbidities affecting the retina, such as diabetes, or those undergoing ophthalmological surgeries. This review also highlights the importance of regular ophthalmology examinations on patients consuming fingolimod both in the initial stages and chronic use. The treatment options for macular edema include nonsteroidal anti-inflammatory drugs, acetazolamide, triamcinolone, ketorolac, corticosteroids, and intravitreal procedures.

## Introduction and background

It is estimated that every five minutes, an individual is diagnosed with multiple sclerosis [1], which is considered the most common inflammatory neurological disorder affecting young adults [2]. In 2016, the worldwide prevalence was 30.1 cases for a population of 100,000 [3]. Multiple sclerosis is a neurological disease that affects the central nervous system and involves various components of the motor, sensory, autonomic, and visual pathways [4]. The inflammation caused by multiple sclerosis leads to lymphatic accumulation, damaging myelin and axons of nerves [4]. The disease presents with symptoms, such as acute unilateral optic neuritis, double vision, facial sensory loss, cerebellar ataxia, nystagmus, sensory loss, Lhermitte’s symptom, asymmetric limb weakness, incontinence, and erectile dysfunction [5]. Both genetic and environmental factors play a role in developing multiple sclerosis [6]. Significant risk factors include contact with birds and fowl; meat consumption of ≥five times a week; vaccination for rubella, measles, varicella, and mumps; right-handedness; family history of multiple sclerosis; thyroiditis; rheumatic diseases; diabetes mellitus; autoimmune diseases with migraines; and comorbidities with autoimmune diseases consistent with migraines [7]. The human leukocyte antigen (HLA) allele DRB1*1501 is primarily linked with the inheritance of multiple sclerosis, with other genes, such as interleukin 2 and 7, CD6 and 58, IRF8, CLEC16A, IL12A, RGS1, PTGERA, TNFAIP3, and TNFRSF1A [6]. The diagnosis of multiple sclerosis is made using the McDonald criteria and is supported by magnetic resonance imaging (MRI) [8]. It involves neurological history, examination, paraclinical laboratory investigation, and diagnostic imaging techniques [8]. Treatment strategies include escalation or early intense therapy [8].

Fingolimod is the first drug approved by the Food and Drug Administration (FDA) to treat multiple sclerosis [9]. A dosage of 0.5 mg consumed once daily is recommended for reducing both clinical symptoms and physical disability caused by the disease [10]. The drug is equally effective in first- and second-line treatments [11]. It is classified as a sphingosine 1-phosphate receptor modulator and is structurally analogous to sphingosine [12]. Initially, the drug undergoes phosphorylation and then interacts with the sphingosine 1-phosphate receptor of both lymphocytes and thrombocytes, resulting in the involution of the receptor and prevention of the cells from leaving secondary lymphoid tissues [12]. Consequently, the lymphocytes are confined to the lymphatic tissue and stopped from accumulating in the central nervous tissue [11]. In animal studies, T cells producing interleukin (IL)-17 have been detected in high amounts in multiple sclerosis and linked to causing damage to neuronal tissue [13]. Fingolimod is essential in the prevention of multiple sclerosis and in diminishing neurological complications [11]. However, the drug is known to cause cardiovascular events, bradycardia, and conduction-delaying effects of the atrioventricular (AV) node [14]. It has also been reported to cause macular edema in patients primarily within four to six months of use [15]. Although precautions should be taken before prescribing fingolimod to patients, including monitoring of blood pressure and other vitals [16], ophthalmological evaluations are also necessary [15].

In Europe, countries, such as Belgium, Italy, and Greece, reported a prevalence of multiple sclerosis ranging from 11 to 282 per 100,000 in women and 10 to 123 per 100,000 in men [17]. Other Latin American countries further from the equator are considered at medium to high risk of developing the disease [18]. Even in countries of high prevalence, such as Pakistan, although numerous therapies are available, not enough data are present on the effects and outcomes of these therapies [19]. By exploring the adverse impacts of fingolimod, one of the primary therapies used in treating multiple sclerosis, better insights can be provided into the drug's treatment, safety, and efficacy. This literature review aims to observe the occurrence, causes, mechanisms, and treatment of fingolimod-associated macular edema in multiple sclerosis.

## Review

Methodology

Two databases, PubMed and Google Scholar, were used to collect relevant articles for this review. The specific keywords used were "fingolimod," "macular edema," "multiple sclerosis," "adverse effects," and "FAME." Each keyword was individually used to search for articles. Medical Subject Headings (MeSH) were also used with the following search strategy: ("Fingolimod"[All Fields] OR "Gilenya"[All Fields]) AND ("Multiple Sclerosis"[All Fields] OR "disseminated sclerosis"[All Fields] OR "encephalomyelitis disseminata"[All Fields]) AND "Macular edema"[All Fields]. This review included only full-text articles in English on multiple sclerosis, fingolimod, and fingolimod-associated macular edema (FAME). All articles not available in full text or not in English were immediately excluded.

Discussion 

Mechanism of Fingolimod and the Role of Sphingosine 1-Phosphate

**Figure 1:**
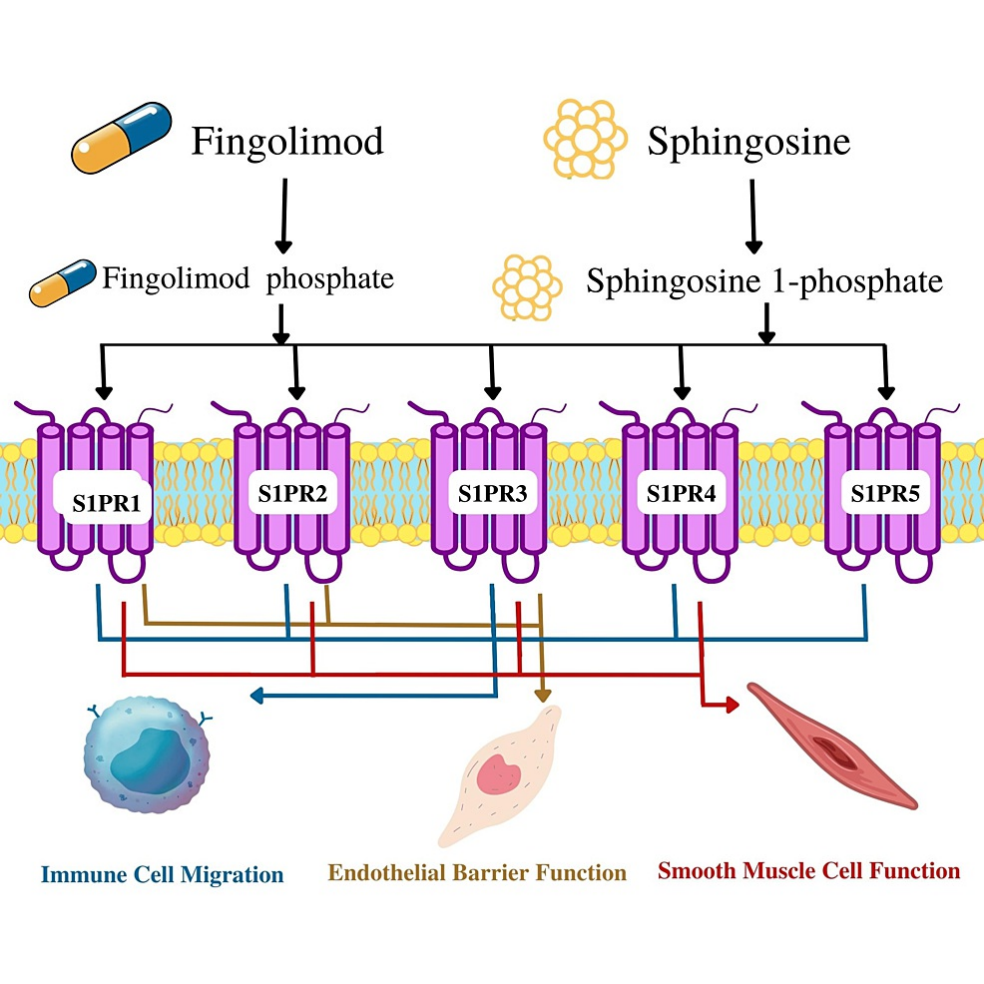
Common functions associated with the sphingosine 1-phosphate receptors (S1PRs) after the action of fingolimod or sphingosine. S1PR1: sphingosine 1-phosphate receptor1 This figure was created by Asma Ashraf Khan.

Fingolimod is classified as a sphingosine, similar to sphingosine 1-phosphate, making up a small portion of sphingolipids [12]. The drug is derived from the chemical modification of a natural compound known as myriocin, acquired from *Isaria sinclairii* [20]. Sphingosine 1-phosphate is an extracellular lipid molecule responsible for signaling through G protein-coupled receptors [12]. The molecule is expressed through the phosphorylation of sphingosine kinases and the involvement of other lyases and phosphatases [21]. It mainly acts on the lymphocytes and endothelium, preventing the accumulation of T and B cells from the lymphatic system, making it a valuable drug for autoimmune diseases [20]. When an antigen is encountered in the blood circulation, sphingosine 1-phosphate receptors (S1PRs) are downregulated on the surface of the lymphocytes, resulting in increased T cell activation and proliferation [21]. There are five subtypes of S1PRs, classified from sphingosine 1-phosphate1 to sphingosine 1-phosphate5 [22]. Sphingosine 1-phosphate1 is responsible for lymphocyte and neural cell migration, cardiovascular and nervous system embryonic development, vascular function, and the role in the endothelial cell barrier [23]. Sphingosine 1-phosphate2 takes part in the vascular tone of vessel walls, endothelial cell function, hearing and balance, and electrical conduction among nerve cells [23]. Similarly, sphingosine 1-phosphate3 participates in endothelial cell function and the migration of nerve cells [24]. Sphingosine 1-phosphate4 takes part in the lymphocyte cell role [24]. Lastly, sphingosine 1-phosphate5 functions in the chemotaxis of natural killer cells and oligodendrocyte activity, as observed in Figure 1 [23]. The numerous effects of sphingosine 1-phosphate on various systems of the body result in a multifocal approach against the targeted disease.

Treatment for Multiple Sclerosis with the Use of Fingolimod

Fingolimod has anti-inflammatory properties, particularly in chronic use [25]. Even with acute use, it has both anticonvulsant and anti-inflammatory effects by decreasing the spike wave and spike amplitude when monitoring the electrophysiological activity [25]. The drug showed a marked decrease in IL-1β, and when paired with diazepam, IL-6 was also slightly decreased, although no change in the tumor necrosis factor (TNF)-α levels was observed [25].

In multiple sclerosis, the dysfunction of the receptor for advanced glycation end products (RAGE) axis plays an essential role in the upregulation of inflammatory processes and neuronal degeneration by the cell signal transduction pathway [26]. With the intake of fingolimod, an increase in the soluble RAGE isoforms known as sRAGE and esRAGE and reduced levels of the RAGE ligand in the serum were observed [27]. Upon MRI investigation, using fingolimod for six to 12 months resulted in decreased relapsing cases of multiple sclerosis and the progression of the disease [27]. A cohort of 2332 patients consuming fingolimod for two and a half years reported decreased relapse rates compared to other drugs, such as teriflunomide (0.18 vs. 0.23; p = 0.05) and dimethyl fumarate (0.20 vs. 0.26; p = 0.01) [28]. The study also observed an increased persistence in the consumption of fingolimod for three months or more and decreased likelihood of patients discontinuing the drug [28]. When monitoring the outcome of fingolimod use on adolescent patients in the age range of 10-17 years who are suffering from relapsing multiple sclerosis and consuming the drug (0.5 mg per day) for two years, a decreased number of relapses and lesions on T2-weighted MRI were also reported [29]. Fingolimod is considered a useful drug for treating relapsing multiple sclerosis because of its physiological and cellular influences on the anti-inflammatory mechanisms of the body.

Fingolimod Adverse Effects

Although fingolimod is a well-known drug against multiple sclerosis, adverse drug effects have been reported among patients. A multicenter double-blinded study observed patients with multiple sclerosis in 18 countries who consumed fingolimod for a duration between 36 months and five years with a dosage of 0.5 mg/day [30]. After a careful analysis, cases of lymphopenia were reported in 6% of the patients, bradycardia in 1%, and first-degree atrioventricular block in another 1% [30]. Serious complications occurred in 25% of the participants, macular edema in 2%, and developed basal cell carcinoma in 4% [30]. At least one adverse effect was reported in 96% of the patients taking fingolimod [30]. The most common adverse effect seen in more than or equal to 5% of the participants was nasopharyngitis, followed by headache and urinary tract infections [30]. Other events included falls, hypertension, increase in alanine aminotransferase and glutamyl gamma transferase levels, back pain, upper respiratory tract infections, arthralgia, constipation, influenza, cough, fatigue, nausea, pain in limbs, dizziness, pyrexia, upper abdominal pain, melanocytic nevi, depression, and insomnia. Cardiac complications were observed in less than 1% of the population, including second-degree atrioventricular block, myocardial infarction, myocardial ischemia, angina pectoris, and secondary hypertension [30]. One case of cystoid macular edema was reported [30], and cases of herpes zoster and dyspnea were also mentioned [30]. Another study observing 107 pediatric patients taking fingolimod reported 16.8% of patients with serious adverse effects [29]. Seizures were reported in six patients of the fingolimod convulsion group [29], and singular cases of agranulocytosis, autoimmune uveitis, bladder spasm, dyspepsia, gastrointestinal necrosis, and vasculitis were detected [29]. A proportion (3.7%) of the patients reported infections of the respiratory and gastrointestinal systems [29]. With the outcomes associated with the chronic use of fingolimod, both physicians and patients should be made aware of the benefits and adverse events linked with fingolimod consumption.

FAME and Changes in Eye Functionality

The drug fingolimod has been observed to have numerous effects on the functionality of the eye. One observational study reported the reduction of neuronal degeneration linked to the consumption of the drug in relapsing-remitting multiple sclerosis [31]. An increase in macular volume was observed after investigating optical coherence tomography reports of patients taking fingolimod and having multiple sclerosis in a longitudinal observational study [32]. A combined group of 30 patients consuming fingolimod for five months had a macular volume increase of 0.025 mm^3^ [32], and in total, 74% of the patients who ingested the drug showed increased macular volume [32]. A decrease in brain volume could result in an increase in retinal volume [32].

**Figure 2:**
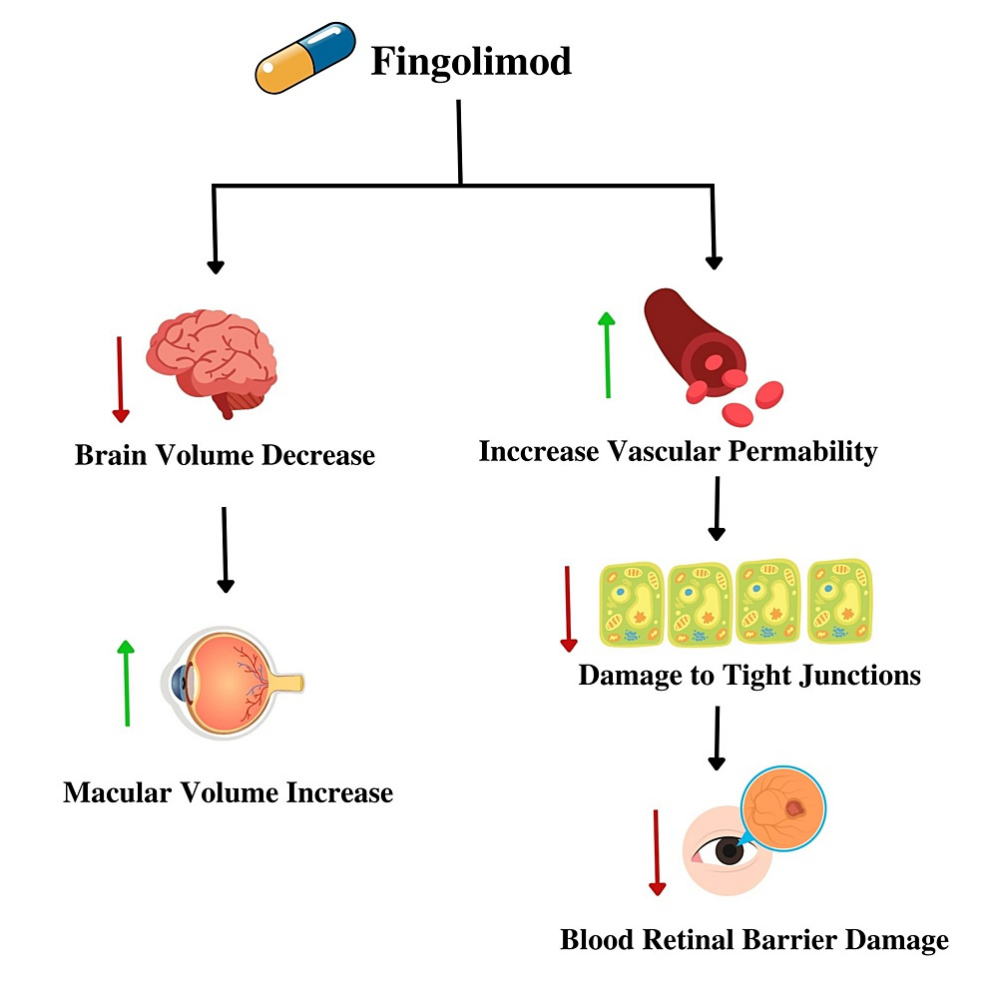
Function of fingolimod on the eye associated with macular edema. This figure was created by Asma Ashraf Khan.

Macular edema is also associated with the fingolimod modulation of S1PRs on the action of tight junctions of the blood-brain barrier and neurons [33]. An increase in vascular permeability could damage the blood-retinal barrier, as observed in Figure 2 [33]. A patient clinically presented with bilateral blurred central vision, painless, and no eye pain with eye movement after 10 days of beginning fingolimod therapy [34]. She had a previous history of optic neuritis affecting only the left eye [34]. The optical coherence tomography revealed bilateral macular edema and an accumulation of fluid between the inner nuclear layer and outer plexiform layer [34]. A decrease in the central foveal thickness and macular volume was seen with the discontinuation of the drug [34]. The relationship between fingolimod and macular edema has been established and observed in relevance to the retinal layer thinning and increase in macular volume.

Fingolimod-Associated Cystoid Macular Edema

The occurrence of cystoid macular edema has been observed and linked to fingolimod use. A 61-year-old female suffering from multiple sclerosis and on chronic use of fingolimod for four years suddenly developed bilateral cystoid macular edema within three weeks of cataract surgery [35]. The blurring of vision was initially reported in the left eye and, after two weeks, proceeded to the right eye [35]. Treatment was started, along with the continuation of fingolimod to prevent the worsening of multiple sclerosis, and the edema was resolved after 13 months [35]. The study highlights the importance of carefully monitoring postoperative patients after cataract surgery, as fingolimod's adverse effects can occur many years later [35]. A 60-year-old female suffering from diabetes also developed cystoid macular edema 10 days after initiation of treatment, and symptoms resolved after discontinuing the drug [36]. Although the patient did not have retinopathy, diabetes-induced microvascular changes in the retina may have contributed to the onset of cystoid macular edema [36]. A 34-year-old female also developed bilateral cystoid macular edema after initiating fingolimod for five days and persistently took the drug for three weeks after visual symptoms arose due to a misdiagnosis. After discontinuing the medication, only the left eye regained normal functionality [37]. The consumption of fingolimod can lead to cystoid macular edema, which often occurs in both eyes and can be related to surgery or comorbidities that previously acted on the retina, such as diabetes. Cystoid edema can occur immediately within a week of treatment or take many years. 

Fingolimod-Associated Risk for Macular Edema

The risk of FAME has been markedly observed in various countries. A study in London observed participants from September 2012 to 2018 for ophthalmological clinical examination and optical coherence tomography [38]. Of the 228 participants, the mean age was 43, 76.8% were females, and 70% were White Caucasians [38]. The initial screening reported a macular edema incidence of 0.88%, but after 637 days, the percentage increased to 1.32% [38]. All cases were resolved within two to 10 months [38]. The study established the importance of continuous ophthalmologic monitoring in patients with fingolimod use, as macular edema can have a delayed onset [38]. A similar analysis of pooled data from three double-blinded randomized clinical trials observed patients aged 18 to 60 consuming 0.5 to 1.25 mg of fingolimod [39]. Of the 2615 patients, 19 patients were diagnosed with macular edema, 0.3% in the 0.5 mg group and 1.2% in the 1.25 mg group [39]. About 68% had the initiation of macular edema between 3 and 4 months of fingolimod use, and only two patients showed macular edema after 12 months of fingolimod use [39]. Moreover, patients with uveitis were at an increased risk of developing macular edema [39]. In Brazil, patients under 18 were identified and followed for 18 months. No adverse events were reported in the 17 participants on ophthalmology exams of the retina [40]. Although the incidence of macular edema is low in participants consuming fingolimod 0.5 mg, patients on chronic use of the drug should undergo regular ophthalmology evaluations to detect early signs of FAME.

FAME Treatment

Multiple treatment options have been observed in different cases according to the patient’s own requirements for FAME. As stated by the FDA guidelines, consistent ophthalmic observation and examinations should be performed after three months of fingolimod therapy to observe any early signs of macular edema [35]. A 42-year-old individual had a sudden onset of macular edema within 24 hours of fingolimod administration [41]. Fundoscopic examination showed increased thickness of the retina [41]. Topical non-steroidal anti-inflammatory drugs were prescribed initially with the discontinuation of fingolimod [41]. After three weeks, when macular edema continued to worsen, acetazolamide was introduced, and the patient recovered [41]. However, once the treatment was stopped, the macular edema rebounded, resulting in steroid administration to the patient [41]. Another case reported in 2017 observed blurring of vision after three months of fingolimod use [42]. No improvement was seen after a month of administration of corticosteroids and non-steroidal anti-inflammatory drops [42]. Fingolimod was then discontinued, improving the patient’s condition and resolving the macular edema reported in the two-month follow-up [42]. A case report in 2022 presented bilateral cystoid macular edema observed in a 61-year-old individual after cataract surgery was treated with a 20 mg sub-Tenon's capsule of triamcinolone acetonide and recovered within 13 months despite continuing fingolimod [35]. Another case reported a 67-year-old female who developed macular edema upon initiation of fingolimod therapy; however, she wanted to continue fingolimod [43]. The patient was administered ketorolac tromethamine and dexamethasone with increased frequency when the deterioration of the left eye continued [43]. Although the patient recovered, tapering off anti-inflammatory drops was unsuccessful, resulting in the cessation of fingolimod [43]. Two cases were reported with an ineffective resolution of macular edema with anti-inflammatory drops, resulting in the use of intravitreal triamcinolone to resolve the edema [44]. The first case noticed a blurring of vision within three weeks of the initiation of fingolimod, but the patient did not discontinue the drug [44]. Despite administering topical steroid drops, no macular improvement was seen in two weeks [44]. She was then given 0.1 mL of intravitreal triamcinolone of 40 mg/mL and noticed symptom improvement in one week, with the restoration of vision observed in the one-month follow-up after treatment [44]. Although multiple treatment options are available for FAME, different approaches have to be used depending on the disease progression of the patient. Moreover, not all cases require the cessation of fingolimod for the resolution of macular edema.

Limitations

Two databases were used to access articles included in this review, and only articles in English were included. Papers available from developing countries were limited. Singular case reports were more abundant in the review due to a limited number of observational studies present. The following limitations could be taken into consideration when interpreting the review.

## Conclusions

This review aimed to observe the mechanisms, risks, and management of fingolimod-associated macular edema. The drug has anti-inflammatory properties through a decrease in cytokines IL-1β, IL-6, spike wave and amplitude, and RAGE ligands. However, the decrease in brain volume and increased vascular permeability result in injuries to tight junctions and increased macular volume, causing damage to the blood-retinal barrier and the occurrence of macular edema. Bilateral cystoid macular edema cases have also been associated with fingolimod use, particularly in people with previous comorbidities associated with retinal damage, such as diabetes. The treatment varies from case to case but may include non-steroidal anti-inflammatory drugs, corticosteroids, ketorolac, and triamcinolone for macular edema resolution, depending on the individual.

This review emphasizes the importance of physicians being aware of the adverse ocular events associated with fingolimod and how careful and regular ophthalmic examinations can help prevent, detect, and manage macular edema. Future studies involving observational studies and randomized control trials of a larger population would be beneficial in observing more clinical associations and patterns.
